# Composite Backward Differentiation Formula for the Bidomain Equations

**DOI:** 10.3389/fphys.2020.591159

**Published:** 2020-12-14

**Authors:** Xindan Gao, Craig S. Henriquez, Wenjun Ying

**Affiliations:** ^1^School of Mathematical Sciences, Shanghai Jiao Tong University, Shanghai, China; ^2^Department of Biomedical Engineering, Pratt School of Engineering, Duke University, Durham, NC, United States

**Keywords:** cardiac, bidomain equations, fully implicit methods, operator splitting, composite backward differentiation formula

## Abstract

The bidomain equations have been widely used to model the electrical activity of cardiac tissue. While it is well-known that implicit methods have much better stability than explicit methods, implicit methods usually require the solution of a very large nonlinear system of equations at each timestep which is computationally prohibitive. In this work, we present two fully implicit time integration methods for the bidomain equations: the backward Euler method and a second-order one-step two-stage composite backward differentiation formula (CBDF2) which is an L-stable time integration method. Using the backward Euler method as fundamental building blocks, the CBDF2 scheme is easily implementable. After solving the nonlinear system resulting from application of the above two fully implicit schemes by a nonlinear elimination method, the obtained nonlinear global system has a much smaller size, whose Jacobian is symmetric and possibly positive definite. Thus, the residual equation of the approximate Newton approach for the global system can be efficiently solved by standard optimal solvers. As an alternative, we point out that the above two implicit methods combined with operator splittings can also efficiently solve the bidomain equations. Numerical results show that the CBDF2 scheme is an efficient time integration method while achieving high stability and accuracy.

## 1. Introduction

The monodomain equations or bidomain equations, consisting of a coupled system of partial differential equations (PDEs) and ordinary differential equations (ODEs), are often used to mathematically model the electrical activity of the heart. The PDEs describe the propagation of the electrical signal through the cardiac tissue and the ODEs describe electrochemical reactions in the cells which are usually nonlinear. Modern myocyte models such as the Ohara-Rudy model and anatomically realistic spatial models enable us to achieve a quantitative understanding of the relationship between molecular function and the integrated behavior of the cardiac myocyte in health and disease, e.g., to predict the clinical risk of drug-induced arrhythmias. It is notable that the cardiac electrical activity typically involves multiple and widely varying scales, which makes the monodomain equations and bidomain equations stiff. As a result, it is computationally expensive to get an accurate solution of the electrical activity of the heart (Tung, 1978; Keener and Sneyd, 1998; Sundnes et al., 2007).

In detailed numerical simulations, the finite element method (Sundnes et al., 2005), finite difference method (Pollard et al., 1992), and finite volume method (Johnston, 2010) have all been used for spatial discretization of the domain. While for temporal discretization, the easiest way is to use explicit methods such as the Runge Kutta method and the forward Euler method since they do not require matrix inversion. However, the timesteps allowed in these methods are generally very small in order to satisfy the requirement of stability (dos Santos et al., 2004; Potse et al., 2006). To overcome the drawbacks of explicit methods, the semi-implicit and Crank-Nicolson methods for solving the PDE component of the bidomain equations are proposed (Keener and Bogar, 1998). But the nonlinear term of the bidomain equations still has restriction on the timesteps for stability (Keener and Bogar, 1998). Although fully implicit methods (Hooke, 1992; Pollard et al., 1992; Dal et al., 2012) preferred for stiff systems can guarantee the stability of the numerical schemes and can use large timesteps, they often have the significant drawback of requiring the solution of a large-scale nonlinear system for diffusion and reaction parts of the bidomain equations at each timestep. For example, a fully implicit parallel Newton-Krylov-Schwarz method for solving the bidomain equations has been proposed in Murillo and Cai (2004). They considered a simple case that the conductivity only varies with the co-ordinate system, i.e., the conductivity tensor is a diagonal matrix in the two-dimensional case. The methods proposed in Murillo and Cai (2004) are difficult to implement for more complex geometries and membrane models.

Operator splitting (Qu and Garfinkel, 1999; Trangenstein and Kim, 2004; Sundnes et al., 2005) is a popular technique that can avoid solving the large nonlinear system of the bidomain equations by splitting the equations into more manageable parts, e.g., splitting the bidomain equations into the linear diffusion and nonlinear reaction parts. An operator splitting method combined with a semi-implicit approximation or a Crank-Nicolson approximation to update the transmembrane and extracellular potentials of the bidomain equations has been proposed in Whiteley (2006) which allows for much larger timesteps.

In this work, we present a backward Euler (BE) and a two-stage composite backward differentiation formula (CBDF2) for solving the cardiac electrical dynamics models. The finite element method is applied for the space discretization. The CBDF2 scheme combines the second-order backward differentiation formula and the BE method with the latter used as fundamental building blocks thus it is fully implicit and easily implementable. We point out that the CBDF2 scheme is unconditionally stable and stiffly accurate for stiff problems, i.e., L-stable. After applying the BE method for the bidomain equations, there are two coupled nonlinear systems: space dependent PDEs and space independent ODEs. The nonlinear elimination method (Ying et al., 2008) is used in this work to solve the space dependent PDEs arising from the fully implicit methods for the bidomain equations. The obtained nonlinear system after elimination has a much smaller size and has a symmetric and positive definite Jacobian when the timestep is small. We solve the nonlinear system by an approximate Newton approach where the residual equations in each Newton iteration can be efficiently solved by standard optimal solvers. As an alternative, we also combine the BE and CBDF2 schemes with Godunov splitting and Strang splitting, respectively, to efficiently solve the bidomain equations. From the numerical results, the CBDF2 scheme allows for timesteps four times larger than that of the BE method while achieving the same high numerical accuracy.

## 2. Materials and Methods

### 2.1. The Model

The bidomain model describing the electrical activity of the heart or a cardiac tissue can be formulated as a system of nonlinear ordinary and partial differential equations (Keener and Sneyd, 1998). Let Ω ⊂ ℝ^*d*^ (*d* > 0) be the bounded computational domain; *t* and **x** ∈ Ω be the temporal and spatial independent variables, respectively; Φ_i_ = Φ_i_(*t*, **x**) and Φ_e_ = Φ_e_(*t*, **x**) be the intracellular and extracellular potentials, respectively; *V*_m_ = *V*_m_(*t*, **x**) = Φ_i_(*t*, **x**)−Φ_e_(*t*, **x**) be the transmembrane potential; **q** represents a set of state variables which can include both gating and ion concentration variables. We consider the bidomain equations of the following form

(1) $$\begin{array}{r} C_{\text{m}}\frac{\partial V_{\text{m}}}{\partial t}+I_{\text{ion}}\left(V_{\text{m}},\text{q}\right)=\frac{1}{\beta }\nabla \cdot \left({\text{D}}_{\text{i}}\nabla \Phi_{\text{i}}\right), \end{array}$$

(2) $$\begin{array}{r} C_{\text{m}}\frac{\partial V_{\text{m}}}{\partial t}+I_{\text{ion}}\left(V_{\text{m}},\text{q}\right)=-\frac{1}{\beta }\nabla \cdot \left({\text{D}}_{\text{e}}\nabla \Phi_{\text{e}}\right)-I_{\text{stim}}, \end{array}$$

(3) $$\begin{array}{r} \frac{\partial \text{q}}{\partial t}=M\left(V_{\text{m}},\text{q}\right), \end{array}$$

for *t* > 0 and **x** ∈ Ω, subject to the homogeneous Neumann boundary condition

$$n\cdot [D_{\text{i}}\nabla \Phi_{\text{i}}]=0\text{ and }n\cdot [D_{\text{e}}\nabla \Phi_{\text{e}}]=0\text{ on }\partial \Omega$$

and one constraint on the intra- and extra-cellular potentials

(4) $$\begin{array}{r} \underset{\Omega }{\int }\Phi_{\text{i}}d\text{x}+\underset{\Omega }{\int }\Phi_{\text{e}}d\text{x}=0. \end{array}$$

Here, *C*_m_ is the membrane capacitance per unit area; β is the surface to volume ratio of the cardiac cells; **D**_i_ and **D**_e_ are the space dependent intracellular and extracellular conductivity tensors, respectively; **n** is the unit outward normal on ∂Ω; *I*_ion_(*V*_m_, **q**) and $M\left(V_{\text{m}},\text{q}\right)$ are two known functions, which are typically nonlinear and describe the electrical dynamics of a cardiac myocyte; *I*_stim_ = *I*_stim_(*t*, **x**) is an extracellular stimulus current. Provided some appropriate initial conditions on the intracellular potential Φ_i_, extracellular potential Φ_e_, and the vector **q** of state variables, the bidomain equations can be uniquely solved. We remark that the bidomain equations for the electrical activity of the heart may take different but equivalent forms (Sundnes et al., 2005; Whiteley, 2006; Vigmond et al., 2008).

### 2.2. The Backward Euler Method

We first introduce the BE method which is used as the fundamental building block of the CBDF2 scheme. Set Δ*t* > 0 as the timestep. Time integration of the bidomain equations by the BE method from time *t*^*n*^ to time *t*^*n*+1^ leads to the semi-discrete bidomain equations

(5) $$\begin{array}{r} C_{\text{m}}\frac{V_{\text{m}}^{n+1}-V_{\text{m}}^{n}}{\Delta t}+I_{\text{ion}}\left(V_{\text{m}}^{n+1},{\text{q}}^{n+1}\right)=\frac{1}{\beta }\nabla \cdot \left({\text{D}}_{\text{i}}\nabla \Phi_{\text{i}}^{n+1}\right), \end{array}$$

(6) $$\begin{array}{r} C_{\text{m}}\frac{V_{\text{m}}^{n+1}-V_{\text{m}}^{n}}{\Delta t}+I_{\text{ion}}\left(V_{\text{m}}^{n+1},{\text{q}}^{n+1}\right)=-\frac{1}{\beta }\nabla \cdot \left({\text{D}}_{\text{e}}\nabla \Phi_{\text{e}}^{n+1}\right)\\ \;-I_{\text{stim}}, \end{array}$$

(7) $$\begin{array}{r} \frac{{\text{q}}^{n+1}-{\text{q}}^{n}}{\Delta t}=M\left(V_{\text{m}}^{n+1},{\text{q}}^{n+1}\right), \end{array}$$

where $\Phi_{\text{i}}^{n}$, $\Phi_{\text{e}}^{n}$, $V_{\text{m}}^{n}$, and **q**^*n*^ are the finite difference approximations of the variables Φ_i_, Φ_e_, *V*_m_, and **q** at time *t*^*n*^, respectively.

To eliminate the state variables **q**^*n*+1^ in Equations (5) and (6), we express the ionic current $I_{\text{ion}}\left(V_{\text{m}}^{n+1},{\text{q}}^{n+1}\right)$ as a function of $V_{\text{m}}^{n+1}$ denoted by

$$I_{\text{ion}}\left(V_{\text{m}}^{n+1}\right)=I_{\text{ion}}\left(V_{\text{m}}^{n+1},{\text{q}}^{n+1}\left(V_{\text{m}}^{n+1}\right)\right)=I_{\text{ion}}\left(V_{\text{m}}^{n+1},{\text{q}}^{n+1}\right),$$

i.e., the vector of state variable **q**^*n*+1^ in Equation (7) can be regarded as a vector value function of the transmembrane potential $V_{\text{m}}^{n+1}$. In our implementation in each timestep, we first give $V_{\text{m}}^{n+1}$ an initial guess, e.g., extrapolated from $V_{\text{m}}^{n}$ and $V_{\text{m}}^{n-1}$, and numerically solve the nonlinear system (7) to obtain the corresponding state variables **q**^*n*+1^ which is substituted into the ionic current function $I_{\text{ion}}\left(V_{\text{m}}^{n+1},{\text{q}}^{n+1}\right)$ to estimate $I_{\text{ion}}\left(V_{\text{m}}^{n+1}\right)$. The value of $I_{\text{ion}}\left(V_{\text{m}}^{n+1}\right)$ is used in the Newton-type iteration formula for solving Equations (5) and (6) to update $V_{\text{m}}^{n+1}$. The above steps are performed iteratively until the residual is less than the tolerance.

We rewrite the Equations (5) and (6) as

(8) $$\begin{array}{r} \mu V_{\text{m}}^{n+1}-\frac{1}{\beta }\nabla \cdot \left({\text{D}}_{\text{i}}\nabla \Phi_{\text{i}}^{n+1}\right)+I_{\text{ion}}\left(V_{\text{m}}^{n+1}\right)=\mu V_{\text{m}}^{n}, \end{array}$$

(9) $$\begin{array}{r} -\mu V_{\text{m}}^{n+1}-\frac{1}{\beta }\nabla \cdot \left({\text{D}}_{\text{e}}\nabla \Phi_{\text{e}}^{n+1}\right)-I_{\text{ion}}\left(V_{\text{m}}^{n+1}\right)=I_{\text{stim}}-\mu V_{\text{m}}^{n}, \end{array}$$

or

(10) $$\begin{array}{r} \left[\begin{array}{cc} \mu -\beta^{-1}\nabla \cdot {\text{D}}_{\text{i}}\nabla & -\mu \\ -\mu & \mu -\beta^{-1}\nabla \cdot {\text{D}}_{\text{e}}\nabla \end{array}\right]\left[\begin{array}{c} \Phi_{\text{i}}^{n+1}\\ \Phi_{\text{e}}^{n+1} \end{array}\right]\\ \;+\left[\begin{array}{c} I_{\text{ion}}\left(V_{\text{m}}^{n+1}\right)\\ -I_{\text{ion}}\left(V_{\text{m}}^{n+1}\right) \end{array}\right]=\left[\begin{array}{c} \mu V_{\text{m}}^{n}\\ I_{\text{stim}}-\mu V_{\text{m}}^{n} \end{array}\right], \end{array}$$

where μ = *C*_m_/Δ*t*.

Assuming that the domain Ω can be partitioned into a quasi-uniform grid if *d* = 2 or a quasi-uniform mesh if *d* = 3, the semi-discrete bidomain Equations (10) can be further discretized by using the continuous piecewise linear finite element method on the grid. Let **M** be the finite element mass matrix; **K**_i_ and **K**_e_ be the finite element stiffness (non-negative) matrices corresponding to the intracellular and extracellular diffusion operators $\left(-\beta^{-1}\nabla \cdot {\text{D}}_{\text{i}}\nabla \right)$ and $\left(-\beta^{-1}\nabla \cdot {\text{D}}_{\text{e}}\nabla \right)$, respectively. Let $\Phi_{\text{i}}^{n}$, $\Phi_{\text{e}}^{n}$, ${\text{V}}_{\text{m}}^{n}$, ${\text{I}}_{\text{ion}}\left(V_{\text{m}}^{n}\right)$, and **I**_stim_ be the column vectors whose entries are the discrete values of the potential and current variables at the corresponding grid nodes. The dimensions of the matrices and vectors are the same and both are equal to the number of nodes in the triangular grid. Using the matrix-vector notation, the discrete finite element bidomain equations now read as

(11) $$\begin{array}{r} \left[\begin{array}{cc} \mu \text{M}+{\text{K}}_{\text{i}} & -\mu \text{M}\\ -\mu \text{M} & \mu \text{M}+{\text{K}}_{\text{e}} \end{array}\right]\left[\begin{array}{c} \Phi_{\text{i}}^{n+1}\\ \Phi_{\text{e}}^{n+1} \end{array}\right]+\left[\begin{array}{c} \text{M}{\text{I}}_{\text{ion}}\left({\text{V}}_{\text{m}}^{n+1}\right)\\ -\text{M}{\text{I}}_{\text{ion}}\left({\text{V}}_{\text{m}}^{n+1}\right) \end{array}\right]\\ \;=\left[\begin{array}{c} \mu \text{M}{\text{V}}_{\text{m}}^{n}\\ \text{M}\left({\text{I}}_{\text{stim}}-\mu {\text{V}}_{\text{m}}^{n}\right) \end{array}\right], \end{array}$$

which is nonlinear and can be solved with an approximate Newton method.

We denote *J*_ion_(*V*_m_) as the derivative of the current function *I*_ion_(*V*_m_) with respect to *V*_m_, i.e.,

$$J_{\text{ion}}\left(V_{\text{m}}\right)=\frac{dI_{\text{ion}}\left(V_{\text{m}}\right)}{dV_{\text{m}}}.$$

Let ${\text{J}}_{\text{ion}}={\text{J}}_{\text{ion}}\left({\text{V}}_{\text{m}}^{n+1}\right)$ be the diagonal matrix with the diagonal entries being the values of $J_{\text{ion}}\left({\text{V}}_{\text{m}}^{n+1}\right)$. Then we have the Jacobian matrix of the nonlinear system (11)

(12) $$\begin{array}{r} \text{A}\equiv \left[\begin{array}{cc} \mu \text{M}+{\text{K}}_{\text{i}} & -\mu \text{M}\\ -\mu \text{M} & \mu \text{M}+{\text{K}}_{\text{e}} \end{array}\right]+\left[\begin{array}{cc} \text{M}{\text{J}}_{\text{ion}} & -\text{M}{\text{J}}_{\text{ion}}\\ -\text{M}{\text{J}}_{\text{ion}} & \text{M}{\text{J}}_{\text{ion}} \end{array}\right]. \end{array}$$

Note that the evaluation of the current function $I_{\text{ion}}\left(V_{\text{m}}^{n+1}\right)$ involves the solution of a local nonlinear system for the vector of state variables **q**^*n*+1^. Since the derivative function $J_{\text{ion}}\left(V_{\text{m}}^{n+1}\right)$ of $I_{\text{ion}}\left(V_{\text{m}}^{n+1}\right)$ may not be directly available, for the convenience of implementation, it is computed approximately by the centered difference

(13) $$\begin{array}{r} J_{\text{ion}}\left(V_{\text{m}}^{n+1}\right)\approx \frac{I_{\text{ion}}\left(V_{\text{m}}^{n+1}+\delta \right)-I_{\text{ion}}\left(V_{\text{m}}^{n+1}-\delta \right)}{2\delta } \end{array}$$

where δ is a small positive parameter, typically on the order of 10^−6^.

Denote $\text{c}={\left(c_{1},c_{2},\ldots ,c_{N}\right)}^{T}$ and $y=\overset{N}{\underset{i=1}{\sum }}c_{i}\psi_{i}$ where {ψ_*i*_} are the piecewise linear basic functions for the finite element method and *N* is the total number of nodes in the quasi-uniform grid or mesh of Ω. Since the conductivity tensors **D**_i_ and **D**_e_ are symmetric and positive definite, for a non-zero vector **c**, the quadratic forms of the stiffness matrices

$${\text{c}}^{T}{\text{K}}_{\text{i}}\text{c}=\frac{1}{\beta }\underset{\Omega }{\int }{\left(\nabla y\right)}^{T}{\text{D}}_{\text{i}}\left(\nabla y\right)d\text{x}$$

and

$${\text{c}}^{T}{\text{K}}_{\text{e}}\text{c}=\frac{1}{\beta }\underset{\Omega }{\int }{\left(\nabla y\right)}^{T}{\text{D}}_{\text{e}}\left(\nabla y\right)d\text{x}$$

are non-negative and vanish if and only if ∇*y*≡0 for any **x** ∈ Ω, which implies that **c** is parallel to (1, 1, …, 1)^*T*^. Therefore, the stiffness matrices **K**_i_ and **K**_e_ are symmetric positive semi-definite and have a rank of *N*−1. We then discuss the property of the Jacobian matrix **A** in Equation (12). Since μ = *C*_m_/Δ*t*, the first part of matrix A in Equation (12) will dominate as Δ*t* approaches zero which is positive semi-definite and has a rank of 2*N*−1. The second part of the matrix **A** is symmetric and negligible compared with the first part when Δ*t* approaches zero. Therefore, with a rank of 2*N*−1, the Jacobian matrix **A** is irreversible, which is consistent with the fact that the solution of the bidomain equations is not unique if the constraint (4) is absent.

To obtain a unique solution, we take the following discrete formula of the constraint (4) as

(14) $$\begin{array}{r} {\text{W}}^{T}\Phi_{\text{i}}+{\text{W}}^{T}\Phi_{\text{e}}=0, \end{array}$$

where $\text{W}={\left(1,1,\ldots ,1\right)}^{T}/\sqrt{N}$ has a dimension of *N*. Then in each approximate Newton iteration, we use the following matrix

(15) $$\begin{array}{r} \text{B}\equiv \text{A}+\left[\begin{array}{c} \text{W}\\ \text{W} \end{array}\right]\left[\begin{array}{cc} {\text{W}}^{T} & {\text{W}}^{T} \end{array}\right] \end{array}$$

to solve the bidomain equations.

Note that the column vector ${\text{W}}_{1}={\left(1,1,\ldots ,1\right)}^{T}$ is an eigenvector corresponding to the eigenvalue 0 of matrix **A**. Denote the other eigenvectors corresponding to the non-zero eigenvalues of matrix **A** by **W**_2_, **W**_3_, … , **W**_2*N*_. Then we have ${\mathbb{R}}^{2N}=\text{span}\left\{{\text{W}}_{1},{\text{W}}_{2},\ldots ,{\text{W}}_{2N}\right\}$, where **W**_1_ is orthogonal to **W**_2_, **W**_3_ … , **W**_2*N*_. Since matrix **A** is non-negative definite, we have ${\text{W}}_{1}^{T}\text{A}{\text{W}}_{1}=0$ and ${\text{W}}_{k}^{T}\text{A}{\text{W}}_{k}>0$, for *k* = 2, …, 2*N*. Obviously, we have

$$\begin{array}{l} {\text{W}}_{1}^{T}\text{B}{\text{W}}_{1}={\text{W}}_{1}^{T}\text{A}{\text{W}}_{1}+{\text{W}}_{1}^{T}{\text{W}}_{1}{\text{W}}_{1}^{T}{\text{W}}_{1}\\ \;={\text{W}}_{1}^{T}{\text{W}}_{1}{\text{W}}_{1}^{T}{\text{W}}_{1}>0 \end{array}$$

and

$$\begin{array}{l} {\text{W}}_{k}^{T}\text{B}{\text{W}}_{k}={\text{W}}_{k}^{T}\text{A}{\text{W}}_{k}+{\text{W}}_{k}^{T}{\text{W}}_{1}{\text{W}}_{1}^{T}{\text{W}}_{k}\\ \;={\text{W}}_{k}^{T}\text{A}{\text{W}}_{k}>0,\text{for}k=2,3,\ldots ,2N. \end{array}$$

Therefore, the modified Jacobian matrix **B** is symmetric and positive definite and the system has a unique solution after adding condition (14).

In principle, we can solve the stabilized system which has a coefficient matrix **B** by a direct method such as the Gauss elimination method or an iterative method such as the Gauss-Seidel iteration and the (preconditioned) conjugate gradient iteration. Because the bidomain equations often need to be discretized on a fine grid with a large number of nodes, the computational work involved with the solution of the linear equations by the above methods may be large and not optimal. To achieve optimal performance, we can apply the V-cycle geometric multigrid method or multigrid preconditioned conjugate gradient method (Wesseling, 1995; Saad, 2003; Ying, 2005) to solve the stabilized linear system in each Newton iteration.

To numerically solve the bidomain equations by the BE method, we give two nonlinear vector-valued functions from Equations (7) and (11) as

(16) $$\begin{array}{r} \text{f}\left(\text{q}\right)\equiv \text{q}-\Delta tM\left({\text{V}}_{\text{m}}^{n+1},\text{q}\right)-{\text{q}}^{n} \end{array}$$

and

(17) $$\begin{array}{r} \text{F}\left({\left(\Phi_{\text{i}},\Phi_{\text{e}}\right)}^{T}\right)\equiv \left[\begin{array}{c} \mu \text{M}{\text{V}}_{\text{m}}^{n}\\ \text{M}\left({\text{I}}_{\text{stim}}-\mu {\text{V}}_{\text{m}}^{n}\right) \end{array}\right]\\ -\left[\begin{array}{cc} \mu \text{M}+{\text{K}}_{\text{i}} & -\mu \text{M}\\ -\mu \text{M} & \mu \text{M}+{\text{K}}_{\text{e}} \end{array}\right]\left[\begin{array}{c} \Phi_{\text{i}}\\ \Phi_{\text{e}} \end{array}\right]-\left[\begin{array}{c} \text{M}{\text{I}}_{\text{ion}}\left({\text{V}}_{\text{m}}\right)\\ -\text{M}{\text{I}}_{\text{ion}}\left({\text{V}}_{\text{m}}\right) \end{array}\right]. \end{array}$$

The system

(18) $$\begin{array}{r} \text{f}\left({\text{q}}^{n+1}\right)=0 \end{array}$$

and

(19) $$\begin{array}{r} \text{F}\left({\left(\Phi_{\text{i}}^{n+1},\Phi_{\text{e}}^{n+1}\right)}^{T}\right)=0 \end{array}$$

from time *t*^*n*^ to *t*^*n*+1^ can be solved by the following detailed Algorithm 1.

**Algorithm 1:** the BE method

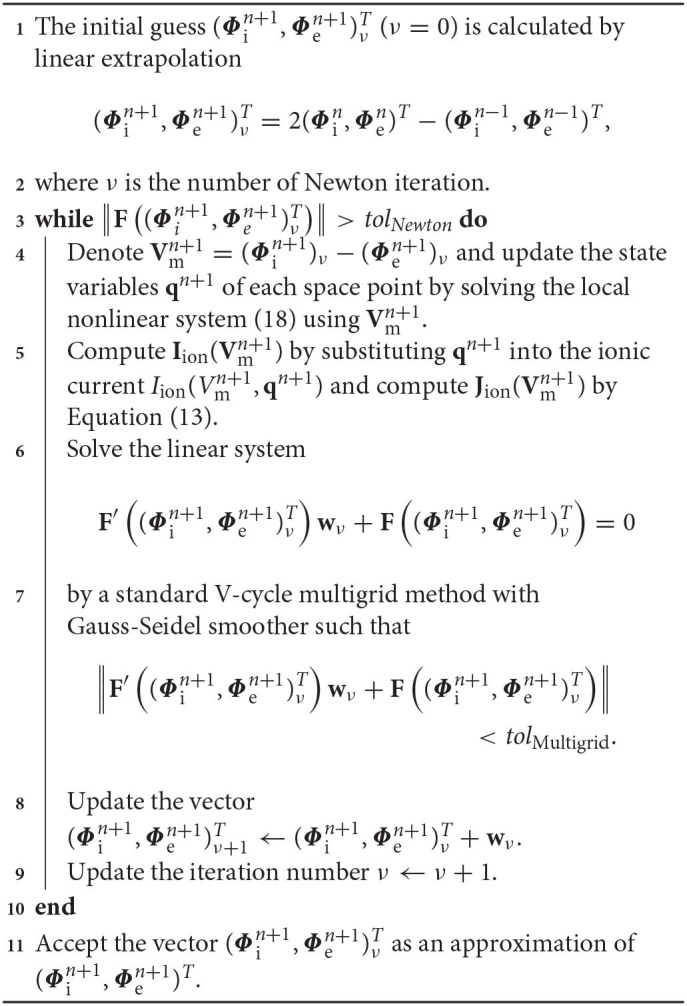

It is worth noting that the updated membrane potential ${\text{V}}_{\text{m}}^{n+1}=\left(\Phi_{\text{i}}^{n+1}-\Phi_{\text{e}}^{n+1}\right)$ has a corresponding updated **q**^*n*+1^, which is computed by solving the nonlinear system of Equation (18), e.g., by the Newton iteration or the damped Newton iteration method.

### 2.3. The Composite Backward Differentiation Formula

We then introduce the CBDF2 scheme (Ying et al., 2008, 2009) to solve the bidomain equations. The CBDF2 scheme has second-order accuracy and is L-stable. It uses the BE method as a fundamental building block. As we have presented how to discretize and solve the bidomain equations with the BE method, the CBDF2 scheme can be readily and easily implemented.

For ease of illustration, we first give the CBDF2 scheme for the abstract form

(20) $$\begin{array}{r} \frac{du\left(t\right)}{dt}=f\left(u\right),\text{for}t>0, \end{array}$$

which can then be straightforwardly extended to the bidomain equations. The CBDF2 scheme for Equation (20) is given by

(21) $$\begin{array}{r} u^{n+\gamma }-\gamma \Delta tf\left(u^{n+\gamma }\right)=u^{n}, \end{array}$$

(22) $$\begin{array}{r} u^{n+1}-\gamma \Delta tf\left(u^{n+1}\right)=\left(2-\frac{1}{\gamma }\right)u^{n}+\left(\frac{1}{\gamma }-1\right)u^{n+\gamma }, \end{array}$$

where the parameter γ takes the value of $1-\sqrt{2}/2$ to obtain a second-order numerical accuracy (Ying et al., 2009). Here, *u*^*n*^ is an approximation of *u*(*t*^*n*^) at time *t*^*n*^ = *nΔt* and *u*^*n*+γ^ is an approximation of *u*(*t*^*n*+γ^) at the intermediate time *t*^*n*+γ^ = *t*^*n*^+γ*Δt*. The right hand side of Equation (22) is an approximation of *u*(*t*^*n*+1−γ^) at time *t*^*n*+1−γ^ = *t*^*n*+1^−γ*Δt* which is extrapolated from the solutions at *t*^*n*^ and *t*^*n*+γ^. Equation (21) is called the first stage and can be regarded as the BE discretization from time *t*^*n*^ to time *t*^*n*+γ^, while Equation (22) is called the second stage and can be regarded as the BE discretization from time *t*^*n*+1−γ^ to time *t*^*n*+1^. The form of the two stages of the CBDF2 scheme are the same as that in the BE temporal discretization except that the timestep is replaced by γ*Δt*. Thus, *u*^*n*+γ^ and *u*^*n*+1^ in Equations (21) and (22) can be computed iteratively as previously discussed in Algorithm 1, i.e., the BE method is the building block of the CBDF2 scheme. To extend the CBDF2 scheme to the bidomain equations, we only need to rewrite the system (1)–(3) to a vector form of Equation (20). The detailed CBDF2 scheme for the bidomain equations is given in the following Algorithm 2.

**Algorithm 2:** the CBDF2 method

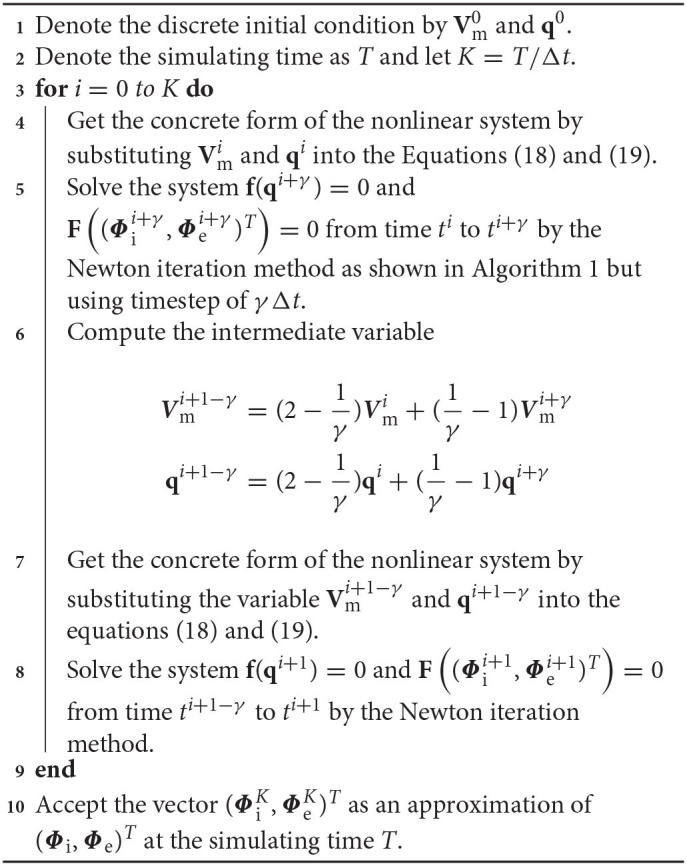

### 2.4. Stability Analysis of The CBDF2 Scheme

We first analyze the stability function of the CBDF2 scheme of a simple ODE

$$\frac{du}{dt}=\lambda u,\text{for}\lambda \in \mathbb{C},$$

which is

$$S\left(z\right)=\frac{1+\left(1-2\gamma \right)z}{{\left(1-\gamma z\right)}^{2}},\text{for}z\in \mathbb{C}.$$

The stability function *S*(*z*) has been proven to be bounded by one for all numbers on the left half complex plane and converge to zero as the real part of the complex number *z* tends to negative infinity with a particular value of the characteristic constant $\gamma =1-\sqrt{2}/2$ (Ying et al., 2008), i.e., the CBDF2 scheme is L-stable.

We then derive the global error of the CBDF2 scheme for Equation (20). Assume the function *f*(*u*) in Equation (20) is Lipschitz continuous in *u* over some feasible domain D, i.e., there exists some constant *L* ≥ 0 such that |*f*(*u*)−*f*(u^*^)| ≤ *L*|*u*−u^*^| for all *u* and u^*^ in D. The local truncation error (LTE) of the CBDF2 scheme (Ying, 2005) has been proven to be

(23) $$\begin{array}{r} E_{\text{CBDF2}}=\left(\frac{1}{6}-\frac{2\gamma^{2}-2\gamma +1}{4\left(1-\gamma \right)}\right){f{}^{\prime }}^{2}f\Delta t^{3}\\ \;+\left(\frac{1}{6}-\frac{1-\gamma }{4}\right)f^{2}f^{\prime \prime }\Delta t^{3}, \end{array}$$

which is on the order of (Δ*t*)^3^.

From the form of the second stage of the CBDF2 scheme (22), we have

(24) $$\begin{array}{r} \frac{u\left(t^{n+1}\right)-\left(\left(2-\frac{1}{\gamma }\right)u\left(t^{n}\right)+\left(\frac{1}{\gamma }-1\right)\tilde{u}\left(t^{n+\gamma }\right)\right)}{\gamma \Delta t}\\ \;=f\left(u\left(t^{n+1}\right)\right)+\frac{E_{\text{CBDF2}}}{\gamma \Delta t}, \end{array}$$

where $\tilde{u}\left(t^{n+\gamma }\right)$ satisfies

(25) $$\begin{array}{r} \frac{\tilde{u}\left(t^{n+\gamma }\right)-u\left(t^{n}\right)}{\gamma \Delta t}=f\left(\tilde{u}\left(t^{n+\gamma }\right)\right). \end{array}$$

Let Equation (25) subtract Equation (21) and we can obtain

(26) $$\begin{array}{r} \left(1-L\gamma \Delta t\right)|{\tilde{e}}^{n+\gamma }|\leq |e^{n}| \end{array}$$

where *e*^*n*^ = *u*(*t*^*n*^)−*u*^*n*^ and ${\tilde{e}}^{n+\gamma }=\tilde{u}\left(t^{n+\gamma }\right)-u^{n+\gamma }$. By subtracting Equation (24) from Equation (22), we have the following inequality

(27) $$\begin{array}{r} \left(1-L\gamma \Delta t\right)|e^{n+1}|\leq |e^{n}|+\left(\frac{1}{\gamma }-1\right)L\gamma \Delta t|{\tilde{e}}^{n+\gamma }|+C_{\text{CBDF2}}\Delta t^{3}\\ \;\leq |e^{n}|+\left(\frac{1}{\gamma }-1\right)\frac{L\gamma \Delta t}{1-L\gamma \Delta t}|e^{n}|\\ \;+C_{\text{CBDF2}}\Delta t^{3}, \end{array}$$

*i*.*e*.,

(28) $$\begin{array}{r} |e^{n+1}|\leq |e^{n}|\frac{1+L\Delta t-2L\gamma \Delta t}{{\left(1-L\gamma \Delta t\right)}^{2}}+\frac{C_{\text{CBDF2}}\Delta t^{3}}{1-L\gamma \Delta t}, \end{array}$$

where $C_{\text{CBDF2}}=|\frac{1}{6}-\frac{2\gamma^{2}-2\gamma +1}{4\left(1-\gamma \right)}|||f^{{}^{\prime }}|{|}_{\infty }^{2}||f|{|}_{\infty }+|\frac{1}{6}-\frac{1-\gamma }{4}|||f|{|}_{\infty }^{2}||f^{{}^{\prime \prime }}|{|}_{\infty }$. When *LΔt* ≤ 1.12, we have $\frac{1+L\Delta t-2L\gamma \Delta t}{{\left(1-L\gamma \Delta t\right)}^{2}}\leq 1+2L\Delta t$ and we can further obtain the global error of the CBDF2 scheme at simulation time *T* as

(29) $$\begin{array}{r} |e^{T/\Delta t}|\leq \left(e^{2LT}-1\right)\frac{C_{\text{CBDF2}}\Delta t^{2}}{2L\left(1-L\gamma \Delta t\right)}. \end{array}$$

Similarly, the LTE of the BE method for the abstract form (20) is Δ*t*^2^*f*′/2, while its global error satisfies

(30) $$\begin{array}{r} |e^{n+1}|\leq \left(1+2L\Delta t\right)|e^{n}|+\frac{\Delta t^{2}||f^{\prime }|{|}_{\infty }}{2\left(1-L\Delta t\right)}, \end{array}$$

and has the form at simulation time *T* as

(31) $$\begin{array}{r} |e^{T/\Delta t}|\leq \left(e^{2LT}-1\right)\frac{\Delta t||f^{\prime }|{|}_{\infty }}{4L\left(1-L\Delta t\right)} \end{array}$$

when *LΔt* ≤ 0.5. Since the restriction for the CBDF2 scheme is *LΔt* ≤ 1.12, the CBDF2 scheme allows a larger timestep than the BE method for the abstract form (20).

## 3. Numerical Results

In this section, we compare the numerical results from the BE and CBDF2 schemes with different spatial step sizes and timestep sizes. The computational domain is partitioned into a quasi-uniform triangular grid in the two-dimensional space and a hexahedral mesh in the three-dimensional space, respectively. A linear approximation is used to obtain the solution on each element. The detailed refinement of a coarser grid to get a finer grid and the numbering principle of the triangular grid are shown in Supplementary Figure 1. Denote the quasi-uniform partition by $T_{\text{h}}$ = {*T*^(1)^, *T*^(2)^, …, *T*^(*M*)^} with *M* being the total number of elements in the partition where *T*^(*i*)^ is the i-th element. Denote *h* as the mesh parameter which measures the maximum of the elements' edges, i.e.,

$$h=\underset{1\leq i\leq M}{\text{max}}\text{edge}\left\{T^{\left(i\right)}\right\}.$$

The transmembrane polarization is driven by an extracellular stimulus which is modeled as a virtual battery with an anode and a cathode in the center of the domain (Vigmond et al., 2008) and is given by

(32) $$\begin{array}{r} I_{\text{stim}}\left(\text{x}\right)=\left\{ \begin{array}{cc} V_{\text{stim}}^{\text{m}}, & \text{if}r<0.1,\\ -V_{\text{stim}}^{\text{m}}, & \text{if}s<0.1, \end{array}\right. \end{array}$$

where $V_{\text{stim}}^{m}$ is a constant, *r* is the distance between **x** and the anode and *s* is the distance between **x** and the cathode.

The operator splitting technique (Qu and Garfinkel, 1999; Trangenstein and Kim, 2004; Sundnes et al., 2005) is also used to efficiently solve the bidomain equations where the linear diffusion part and the nonlinear reaction part are solved separately. To be specific, the Godunov splitting combined with the BE method and the Strang splitting combined with the CBDF2 scheme are used to solve the bidomain equations (see Supplementary Material for details). The linear system obtained from discretizing the diffusion part is solved by the standard multigrid V-cycle with Gauss-Seidel smoother. Notice that the coefficient matrix of the discrete linear system obtained from the operator splitting does not contain the evaluation of the derivative function $J_{\text{ion}}\left(V_{\text{m}}^{n+1}\right)$, i.e., the second part of matrix **A**.

In the numerical simulation of the above methods, the membrane capacitance per unit area and the surface-to-volume ratio are set as *C*_m_ = 1 (units: μF/cm^2^) and β = 1, 000 (units: cm^−1^), respectively. Other constant parameters are set as $tol_{\text{Newton}}=10^{-7}$, $tol_{\text{Multigrid}}=10^{-7}$, and the number of smoothing iterations ν_Multigrid_ = 6. The absolute tolerance in the Newton iteration method for solving the nonlinear system (18) is chosen to be 10^−10^. The parameter δ in Equation (13) is selected to be 10^−6^ for all simulations. The units for the time steps, spatial steps, and voltage are milliseconds (ms), centimeters (cm), and millivolts (mV), respectively. The unit of conductivity is millisiemens per centimeter (mS/cm^−1^). The fully implicit integration methods are implemented with custom codes written in C++ and the numerical simulations are all performed on a 3.6 GHz computer with an Intel Core i3-4160 CPU.

We should mention that the computational domain is not limited to the regular areas. For example, circular regions can be divided into a quasi-uniform triangular grid by the same principle as shown in Supplementary Figure 8 and a solid sphere can be partitioned into a quasi-uniform tetrahedral mesh (Liu and Joe, 1996; Everett, 2010). The additional numerical results solved on a circle and sphere are shown in Supplementary Figures 9–13.

### 3.1. Numerical Example in Two-Dimensional Space

For the numerical simulations of the bidomain equations in the two-dimensional space, we model the square consisting of fibers which rotate counterclockwise around the point **c** = (*c*_0_, *c*_1_) = (−0.5, −0.5). The conductivity tensor is space dependent with unequal anisotropy ratios: in the intracellular space, it is 12.0 along the fiber and 2.0 perpendicular to the fiber; in the extracellular space, it is 8.0 along the fiber and 4.0 perpendicular to the fiber. The conductivity tensors **D**_i_(**x**) and **D**_e_(**x**) with **x** = (*x*_0_, *x*_1_) can be expressed by

$${\text{D}}_{\text{i}}\left(\text{x}\right)=\sigma_{\text{i}}^{\text{l}}{\text{e}}_{\text{l}}{\text{e}}_{\text{l}}^{T}+\sigma_{\text{i}}^{\text{n}}{\text{e}}_{\text{n}}{\text{e}}_{\text{n}}^{T}\text{and}{\text{D}}_{\text{e}}\left(\text{x}\right)=\sigma_{\text{e}}^{\text{l}}{\text{e}}_{\text{l}}{\text{e}}_{\text{l}}^{T}+\sigma_{\text{e}}^{\text{n}}{\text{e}}_{\text{n}}{\text{e}}_{\text{n}}^{T},$$

where $\sigma_{\text{i}}^{\text{l}}=12$, $\sigma_{\text{i}}^{n}=2$, $\sigma_{\text{e}}^{\text{l}}=8$, $\sigma_{\text{e}}^{\text{n}}=4$, ${\text{e}}_{\text{l}}={\left(\text{sin}\theta ,\text{cos}\theta \right)}^{T}$, and ${\text{e}}_{\text{n}}={\left(-\text{cos}\theta ,\text{sin}\theta \right)}^{T}$ with $\text{cos}\theta =\frac{x_{0}-c_{0}}{\left|\text{x}-\text{c}\right|}$ and $\text{sin}\theta =\frac{c_{1}-x_{1}}{\left|\text{x}-\text{c}\right|}$. The positions of the anode and cathode are set as (0.875, 1.0) and (1.125, 1.0), respectively.

In this work, we first use a variant of the FitzHugh-Nagumo (Vfhn) model as the membrane dynamics which is governed by

(33) $$\begin{array}{r} I_{\text{ion}}\left(V_{\text{m}},q\right)=GV_{\text{m}}\left[\left(1-\frac{V_{\text{m}}}{V_{\text{th}}}\right)\left(1-\frac{V_{\text{m}}}{V_{\text{peak}}}\right)+q\right]\\ M\left(V_{\text{m}},q\right)=\gamma \left(\frac{\alpha V_{\text{m}}}{V_{\text{peak}}}-q\right) \end{array}$$

with the parameters set as *V*_peak_ = 1, *G* = 20, *V*_th_ = 0.125, α = 3, and γ = 1. The gating and ion concentration variables **q**, the intracellular potential Φ_i_, and the extracellular potential Φ_e_ are set to be at rest at time *t* = 0.

**Figure 1:**
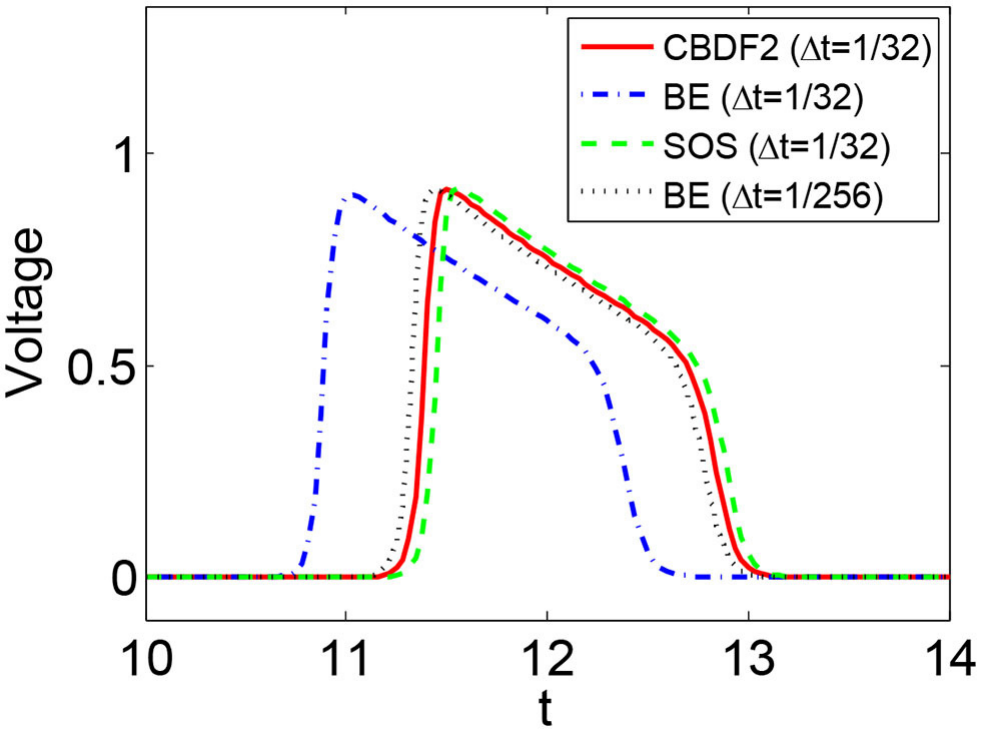
The membrane voltage trajectories obtained from different methods with timestep size Δ*t* = 1/32 and spatial size *h* = 1/64. A high precision solution is obtained from the BE method with a small timestep size Δ*t* = 1/256 and spatial size *h* = 1/256. The membrane model is the Vfhn model.

**Figure 2:**
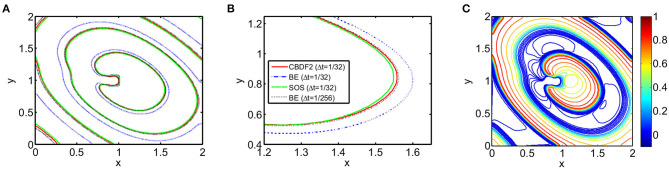
The isopotential lines *V*_m_ = 0.1 at time *T* = 8.75 from different methods with timestep size Δ*t* = 1/32 and spatial size *h* = 1/64. A high precision solution is obtained from the BE method with a small timestep size Δ*t* = 1/256 and spatial size *h* = 1/256. **(A)** The isopotential lines in the entire computational domain. **(B)** A zoomed region of **(A)** to clearly display the different isopotential lines. **(C)** Contours for the transmembrane potential *V*_m_ of an anisotropic bidomain equation at *T* = 8.75 from the high precision solution.

**Figure 3:**
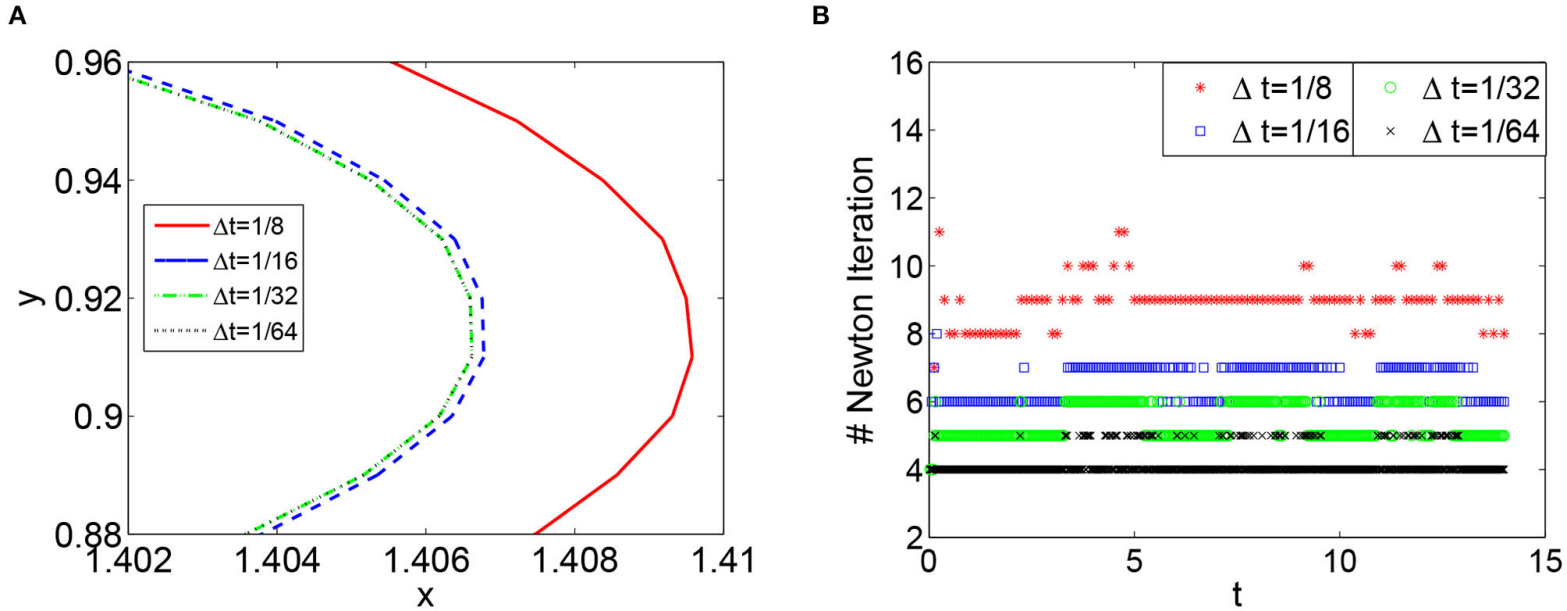
**(A)** The isopotential lines *V*_m_ = 0.1 obtained from the CBDF2 scheme using different timesteps in the area around the point (1.4, 1) at *T* = 8. **(B)** The number of Newton iteration in each simulation using different timesteps sizes. The spatial step size in **(A,B)** is *h* = 1/64.

We first discuss the numerical performance of the CBDF2 scheme. Using the same timestep size Δ*t* = 1/32 and spatial step size *h* = 1/64, we compare the CBDF2 scheme with the other different time integration methods. A high precision solution obtained from the BE method using a very small timestep size Δ*t* = 1/256 and spatial step size *h* = 1/256 is set as a benchmark. As shown in Figure 1, the CBDF2 scheme using a large timestep can obtain a highly accurate trajectory of the membrane voltage compared with the benchmark while that obtained from the BE method using a large timestep is inaccurate. Besides, the Strang operator splitting (SOS) combined with the CBDF2 scheme is stable but less accurate than the CBDF2 scheme in the membrane voltage trajectory. We point out that the Godunov operator splitting combined with the BE method is unstable using the timestep size Δ*t* = 1/32 and spatial step size *h* = 1/64. Figure 2 shows the isopotential lines *V*_m_ = 0.1 at time *T* = 8.75 for the bidomain simulations from the above methods, while Figure 3 shows the isopotential lines *V*_m_ = 0.1 and the Newton iteration number obtained from the CBDF2 scheme using different timesteps.

We next study the convergence of the four different time schemes, i.e., the BE method, the CBDF2 scheme, the Godunov splitting combined with the BE method, and the Strang splitting combined with the CBDF2 scheme with the Vfhn membrane model. Comparing with the high precision numerical solution obtained from the BE method with a quite small timestep size Δ*t* = 1/512 and spatial step size *h* = 1/512, we compute the errors of the membrane voltage which is summed over the whole space at the discrete times. To be specific, we use the scaled discrete *l*^2^-norm of a vector $\text{v}={\left(v_{1},v_{2},\cdots \;,v_{d}\right)}^{T}\in {\mathbb{R}}^{d}$ defined as

$$||\text{v}|{|}_{l^{2}}=\sqrt{\frac{1}{d}\overset{d}{\underset{i=1}{\sum }}v_{i}^{2}}$$

to compute the error of voltage summed over the whole domain, i.e., ${\text{e}}^{\left(\text{x}\right)}={\left(e({\text{x}}_{1}),e({\text{x}}_{2}),\cdots ,e({\text{x}}_{N})\right)}^{T}$. By halving both the timestep and spatial step size, we can obtain a first-order convergence of the BE method and the Godunov splitting combined with the BE method and a second-order convergence of the CBDF2 scheme and the Strang splitting combined with the CBDF2 scheme, respectively, as shown in Table 1.

**Table 1:** Errors of membrane potentials in the scaled discrete *l*^2^-norm of four different time integration methods.

	**Δ*t* = *h* = 1/32**	**Δ*t* = *h* = 1/64**	**Δ*t* = *h* = 1/128**	**Δ*t* = *h* = 1/256**
BE	1.66e-01	9.98e-02	4.92e-02	1.72e-02
Order	–	0.7	1.0	1.5
CBDF2	1.05e-01	3.08e-02	8.20e-03	1.82e-03
Order	–	1.8	1.9	2.2
Godunov	*	1.14e-01	6.13e-02	2.73e-02
Order	*	–	0.9	1.2
Strang	1.49e-01	4.15e-02	1.25e-02	4.15e-03
Order	–	1.8	1.7	1.6

The computational domain is a rectangle [0, 2]^2^ and the total run time is T = 2. The membrane model is the Vfhn model and asterisk(*) indicates overflow error.

We further consider two more realistic membrane models, i.e., the DiFrancesco and Noble (DFN) model (DiFrancesco and Noble, 1985; Cabo and Barr, 2006) and the Courtemanche et al. (CRN) model (Courtemanche et al., 1998) which have 15 and 20 state variables other than the transmembrane potential, respectively. In each simulation, the state variables **q** are all assumed to be at rest at time *t* = 0 and the initial condition of the membrane potential *V*_m_ is given by

$$V_{\text{m}}\left(\text{x},t=0\right)=\frac{100}{1+\text{exp}\left(200\left(\sqrt{x_{1}^{2}+x_{2}^{2}}-0.2\right)\right)}-80.$$

The iso-contours and trajectories of the membrane voltage obtained from the CBDF2 scheme using the more realistic DFN and CRN models in the two-dimensional space is shown in Supplementary Figure 6.

We study the stability of the four different time integration methods with the DFN membrane model. The simulations for the bidomain equation with the DFN model use timestep sizes of 1/8, 1/16, 1/32, 1/64, 1/128, and spatial step sizes of 1/128. As shown in Table 2, the CBDF2 scheme is stable for the timestep sizes ≤ 1/8 while the BE method is stable for timestep sizes ≤ 1/32. The Strang operator splitting together with the CBDF2 scheme is stable for the timestep sizes less than or equal to 1/8 while the Godunov operator splitting together with the BE method is stable for the timestep sizes less than or equal to 1/16. The numerical results show that the CBDF2 scheme has better stability compared with the BE method which is consistent with the stability analysis in section 2.4.

**Table 2:** The comparison of CPU times (minutes) and the error of membrane voltage of the four methods.

	**BE**		**CBDF2**		**Godunov**		**Strang**	
***Δt***	**CPU**	**$||{\text{e}}^{\left(\text{x}\right)}{\left|\right|}_{l^{2}}$**	**CPU**	**$||{\text{e}}^{\left(\text{x}\right)}{\left|\right|}_{l^{2}}$**	**CPU**	**$||{\text{e}}^{\left(\text{x}\right)}{\left|\right|}_{l^{2}}$**	**CPU**	**$||{\text{e}}^{\left(\text{x}\right)}{\left|\right|}_{l^{2}}$**
1/128	20.27	8.92e-03	50.96	7.10e-03	15.27	9.97e-03	34.23	8.19e-03
1/64	16.79	1.07e-02	30.36	8.09e-03	9.32	1.43e-02	19.63	1.35e-02
1/32	15.75	3.05e-02	21.54	8.33e-03	6.61	3.96e-02	11.21	1.52e-02
1/16	*	*	14.79	8.60e-03	4.88	9.07e-02	7.36	1.57e-02
1/8	*	*	13.75	9.78e-03	*	*	4.97	2.59e-02

The computational domain is a rectangle [0, 1]^2^, the total run time is T = 10, and the spatial step size is 1/128. The membrane model is the DFN model and asterisk(*) indicates overflow error.

As shown in Table 2, the Godunov splitting combined with the BE method is less stable than the Strang splitting combined with the CBDF2 scheme. A possible reason is that the BE method is less stable than the CBDF2 scheme. To fairly compare the stability of the Godunov splitting and Strang splitting, we further perform numerical experiments using Strang splitting combined with the BE method which allows using a much larger time step (maximum Δ*t* ≈ 0.20) than the Godunov splitting combined with the BE method. Our experiments indicate that the Strang splitting has better stability than the Godunov splitting for the bidomain equations.

To compare the numerical accuracy of each scheme, a high precision solution is obtained from the BE method with a very small timestep Δ*t* = 1/1, 024 and spatial step size *h* = 1/128. As shown in Table 2, the CBDF2 scheme using a large timestep Δ*t* = 1/8 is more accurate and efficient compared with the BE method using a small timestep 1/64. Besides, the CBDF2 scheme, combined with the nonlinear elimination method, has better accuracy than the CBDF2 scheme combined with Strang operator splitting although taking a bit more time as shown in Table 2. Note that the errors in Table 2 do not convergence to zero as the timestep size is reduced. This is because the errors depend on both the timestep size and spatial step size which have a lower bound when using a fixed spatial step size.

We emphasize that the computational domain is not limited to the regular areas such as rectangle regions. Numerical results solved on a circular region which is partitioned into a quasi-uniform triangular grid (Liu and Joe, 1996; Everett, 2010) are given in Supplementary Figures 9, 10. The performance of the CBDF2 scheme is as well as that solved on the rectangle region.

### 3.2. Numerical Example in Three-Dimensional Space

The numerical methods introduced in this work can be straightforwardly extended to the case of three-dimensional space. For ease of illustration, we first simulate the bidomain equations with the Vfhn membrane on a unit cube **x** ∈ [0, 1]^3^ ($\text{x}={\left(x_{1},x_{2},x_{3}\right)}^{T}$). The extracellular stimulus in the three-dimensional space is given by applying a virtual battery with an anode and a cathode located at (0.375, 0.5, 0.5) and (0.625, 0.5, 0.5) in the center of the domain and is given by

(34) $$\begin{array}{r} I_{\text{stim}}\left(\text{x}\right)=\left\{ \begin{array}{cc} 6.0, & \text{if}r<0.1,\\ -6.0, & \text{if}s<0.1, \end{array}\right. \end{array}$$

where *r* is the distance between **x** and the anode and *s* is the distance between **x** and the cathode. The computational domain in three-dimensional space is partitioned into a quasi-uniform hexahedron mesh. We use a linear approximation to the solution on each element. The parameters, tolerances, and modules are set the same as those noted previously in this work.

In the case of three-dimensional space, the axially isotropic conductivity tensors are formulated as

$$\begin{array}{l} {\text{D}}_{\text{i}}=\sigma_{\text{i}}^{\text{n}}\text{I}+\left(\sigma_{\text{i}}^{\text{l}}-\sigma_{\text{i}}^{\text{n}}\right){\text{e}}_{\text{l}}{\text{e}}_{\text{l}}^{T}\text{and}{\text{D}}_{\text{e}}=\sigma_{\text{e}}^{\text{n}}\text{I}+\left(\sigma_{\text{e}}^{\text{l}}-\sigma_{\text{e}}^{\text{n}}\right){\text{e}}_{\text{l}}{\text{e}}_{\text{l}}^{T}, \end{array}$$

where **I** is the 3 × 3 identity matrix, $\sigma_{\text{i}}^{\text{l}}=12$, $\sigma_{\text{i}}^{\text{n}}=2$, $\sigma_{\text{e}}^{\text{l}}=8$, and $\sigma_{\text{e}}^{\text{n}}=4$. The readers can refer to Neu and Krassowska (1993) and Ying (2005) for more details of the conductivity tensors.

The iso-contour and iso-surface plots of the membrane potential simulated on a unit cube are shown in Supplementary Figures 2–5. Additional numerical results obtained from simulating the bidomain equations in a solid sphere are shown in Supplementary Figures 11–13.

We then consider the DFN and CRN models in three-dimensional space. In each simulation, the state variables **q** are all assumed to be at rest at time *t* = 0 and the initial condition of the membrane potential *V*_m_ is given by

$$V_{\text{m}}\left(\text{x},t=0\right)=\frac{100}{1+\text{exp}\left(200\left(\sqrt{x_{1}^{2}+x_{2}^{2}+x_{3}^{2}}-0.2\right)\right)}-80.$$

The iso-contours and trajectories of the membrane voltage obtained from the CBDF2 scheme using the more realistic DFN and CRN models in the three-dimensional space are shown in Supplementary Figure 7.

## 4. Discussion

In this work, we have presented two fully implicit methods, the BE and CBDF2 schemes, to numerically simulate the bidomain equations arising from modeling the electrical activity of the heart. The second-order CBDF2 scheme is L-stable for the stiff problems and uses the BE method as building blocks. The CBDF2 scheme has better accuracy and efficiency using a large timestep Δ*t* = 1/8 compared with the BE method using a small timestep Δ*t* = 1/64. When the error of membrane voltage for the CBDF2 and BE schemes are approximately the same, the CBDF2 scheme allows for timestep an order of magnitude larger than that used in the BE method. A further advantage of the CBDF2 scheme is that it allows temporal adaptivity to speedup, i.e., it can use a small timestep during the action potential upstroke period (stiff region) and a much larger timestep when the electrical activity is slowly varying (non-stiff region) (Ying, 2005; Whiteley, 2007). The CBDF2 scheme satisfies the requirement of stability, accuracy, and efficiency well which enable us to achieve a quantitative understanding of the relationship between molecular function and the integrated behavior of the cardiac myocyte in health and disease more effectively, e.g., to predict the clinical risk of drug-induced arrhythmias.

When using fully implicit methods to solve the bidomain equations, the obtained nonlinear systems are generally very large. We use a variant of the nonlinear elimination method (Lanzkron and Rose, 1997) to reduce the size of the system which includes the evaluation of the Jacobian matrix for each timestep. The spatially and temporally discretized nonlinear system (11) is solved with an approximate Newton method where the coefficient matrix of the residual system is symmetric and possibly positive definite consisting of the stiffness matrix, mass matrix, and the derivative function *J*_ion_. The linear residual equation can be efficiently solved by standard optimal solvers such as the V-cycle multigrid method (Saad, 2003). The derivative function *J*_ion_ is time dependent and can be numerically evaluated with sufficient accuracy.

The computational domains in this work are square and cube (see Supplementary Figures 9–13 for results simulated on circle and sphere), but we emphasize that the CBDF2 scheme can be applied to more complicated computational domains since the generation rules of the finite element mesh in both two- and three-dimensional space do not depend on the particularity of the square or cube. The generation rules are applicable to more complex computational domains such as the realistic human left ventricle model (Cai et al., 2015) which will be presented in our future work.

In addition, the CBDF2 scheme using an adaptive mesh refinement algorithm can further improve the computational efficiency (Trangenstein and Kim, 2004; Ying, 2005; Whiteley, 2007). For the parallel implementation, the fully implicit methods proposed here leads to a full space independent system which is highly localized. As the inter-processor communication for updating the state variables by solving Equation (7) will be much less, the speedup for the simulations will be impressive when the membrane model including tens of or more state variables. In future works, we will consider more realistic computational domains such as the human left ventricle domain (Cai et al., 2015) combined with more realistic membrane models such as the Tusscher-Noble-Noble-Panfilov model (ten Tusscher et al., 2004) which can potentially help study new drugs and methods of treatment.

## Data Availability Statement

The raw data supporting the conclusions of this article will be made available by the authors, without undue reservation.

## Author Contributions

XG and WY: conception, design, code implementation, data analysis, and drafting. CH: design and critical revision. All authors contributed to the article and approved the submitted version.

## Conflict of Interest

The authors declare that the research was conducted in the absence of any commercial or financial relationships that could be construed as a potential conflict of interest.
