# Supplementary material for: Composite Backward Differentiation Formula for the Bidomain Equations
Source: Front Physiol. 2020 Dec 14;11:591159. doi: 10.3389/fphys.2020.591159 (PMC7767930; doi:10.3389/fphys.2020.591159)
Supplement: Supplementary file 1 [file Data_Sheet_1.pdf]

Supplementary Material

1 OPERATOR SPLITTING

Operator splitting (Qu and Garfinkel, 1999; Sundnes et al., 2005; Trangenstein and Kim, 2004) is a popular technique that can avoid solving the large nonlinear systems for the bidomain equations by splitting the equations into more manageable parts. In this work, the bidomain equations

$$C_{\mathrm{m}}\frac{\partial V_{\mathrm{m}}}{\partial t} + I_{\mathrm{ion}}(V_{\mathrm{m}}, \mathbf{q}) = \frac{1}{\beta}\nabla \cdot (\mathbf{D}_{\mathrm{i}}\nabla \varPhi_{\mathrm{i}}), \tag{S1}$$

$$C_{\mathrm{m}}\frac{\partial V_{\mathrm{m}}}{\partial t} + I_{\mathrm{ion}}(V_{\mathrm{m}}, \mathbf{q}) = -\frac{1}{\beta}\nabla \cdot (\mathbf{D}_{\mathrm{e}}\nabla \varPhi_{\mathrm{e}}) - I_{\mathrm{stim}}, \tag{S2}$$

$$\frac{\partial \mathbf{q}}{\partial t} = \mathcal{M}(V_{\mathrm{m}}, \mathbf{q}), \tag{S3}$$

are split into the linear diffusion and nonlinear reaction parts respectively as

$$C_{\mathrm{m}}\frac{\partial V_{\mathrm{m}}}{\partial t} = \frac{1}{\beta}\nabla \cdot (\mathbf{D}_{\mathrm{i}}\nabla \varPhi_{\mathrm{i}}), \tag{S4}$$

$$C_{\mathrm{m}}\frac{\partial V_{\mathrm{m}}}{\partial t} = -\frac{1}{\beta}\nabla \cdot (\mathbf{D}_{\mathrm{e}}\nabla \varPhi_{\mathrm{e}}) - I_{\mathrm{stim}}, \tag{S5}$$

and

$$C_{\mathrm{m}}\frac{\partial V_{\mathrm{m}}}{\partial t} = -I_{\mathrm{ion}}(V_{\mathrm{m}}, \mathbf{q}), \tag{S6}$$

$$\frac{\partial \mathbf{q}}{\partial t} = \mathcal{M}(V_{\mathrm{m}}, \mathbf{q}). \tag{S7}$$

1.1 Godunov Operator Splitting

To implement the Godunov operator splitting combined with the backward Euler (BE) method, the bidomain equations from time t^n to t^{n+1} are reduced to the semi-discrete linear diffusion system

$$C_{\mathrm{m}}\frac{V_{\mathrm{m}}^{n+1} - V_{\mathrm{m}}^{n}}{\Delta t} = \frac{1}{\beta}\nabla \cdot (\mathbf{D}_{\mathrm{i}}\nabla \varPhi_{\mathrm{i}}^{n+1}), \tag{S8}$$

$$C_{\mathrm{m}}\frac{V_{\mathrm{m}}^{n+1} - V_{\mathrm{m}}^{n}}{\Delta t} = -\frac{1}{\beta}\nabla \cdot (\mathbf{D}_{\mathrm{e}}\nabla \varPhi_{\mathrm{e}}^{n+1}) - I_{\mathrm{stim}}, \tag{S9}$$

where the transmembrane potential V_{m} is defined as the difference of intra- and extra-cellular potentials, *i.e.*, $V_{\mathrm{m}} = \varPhi_{\mathrm{i}} - \varPhi_{\mathrm{e}}$. Using the advanced data from the diffusion system as the initial

value, the finite difference system for the nonlinear reaction part is given by

$$C_\mathrm{m}\frac{V_\mathrm{m}^{n+1}-V_\mathrm{m}^{n}}{\Delta t} = -I_\mathrm{ion}(V_\mathrm{m}^{n+1},\mathbf{q}^{n+1}), \tag{S10}$$

$$\frac{\mathbf{q}^{n+1}-\mathbf{q}^{n}}{\Delta t} = \mathcal{M}(V_\mathrm{m}^{n+1},\mathbf{q}^{n+1}). \tag{S11}$$

The linear diffusion system in Equations (S8) and (S9) can be further discretized by using continuous piecewise linear finite element method on the grid, and can be solved by optimal solvers such as V-cycle geometric multigrid method. The nonlinear system in Equations (S10) and (S11) is space independent and can be solved by the Newton iteration method.

1.2 Strang Operator Splitting

We also use a variant of the Strang operator splitting combined with a second-order one-step two-stage composite backward differentiation formula (CBDF2) to solve the bidomain equations from time t^n to t^{n+1}, which consists of the following three steps.

step 1: Integrate the nonlinear reaction part by the forward Euler method

$$C_\mathrm{m}\frac{W_\mathrm{m}^{n}-V_\mathrm{m}^{n}}{\Delta t/2} = -I_\mathrm{ion}(V_\mathrm{m}^{n},\mathbf{q}^{n}), \tag{S12}$$

$$\frac{\mathbf{q}^{n+1/2}-\mathbf{q}^{n}}{\Delta t/2} = \mathcal{M}(V_\mathrm{m}^{n},\mathbf{q}^{n}), \tag{S13}$$

to compute W_m^n and $\mathbf{q}^{n+1/2}$.

step 2: Integrate the linear diffusion part by the CBDF2 scheme

- integrate by the BE method from time t^n to $t^{n+\gamma}$

$$C_\mathrm{m}\frac{W_\mathrm{m}^{n+\gamma}-W_\mathrm{m}^{n}}{\gamma\Delta t} = \frac{1}{\beta}\nabla\cdot(\mathbf{D}_\mathrm{i}\nabla\Phi_\mathrm{i}^{n+\gamma}), \tag{S14}$$

$$C_\mathrm{m}\frac{W_\mathrm{m}^{n+\gamma}-W_\mathrm{m}^{n}}{\gamma\Delta t} = -\frac{1}{\beta}\nabla\cdot(\mathbf{D}_\mathrm{e}\nabla\Phi_\mathrm{e}^{n+\gamma}) - I_\mathrm{stim}. \tag{S15}$$

- extrapolate the value of W_m at time $t^{n+1-\gamma}$

$$W_\mathrm{m}^{n+1-\gamma} = (2-\frac{1}{\gamma})W_\mathrm{m}^{n} + (\frac{1}{\gamma}-1)W_\mathrm{m}^{n+\gamma}. \tag{S16}$$

- integrate by the BE method from time $t^{n+1-\gamma}$ to t^{n+1}

$$C_{\mathrm{m}}\frac{W_{\mathrm{m}}^{n+1}-W_{\mathrm{m}}^{n+1-\gamma}}{\gamma\Delta t}=\frac{1}{\beta}\nabla\cdot(\mathbf{D}_{\mathrm{i}}\nabla\Phi_{\mathrm{i}}^{n+1}), \tag{S17}$$

$$C_{\mathrm{m}}\frac{W_{\mathrm{m}}^{n+1}-W_{\mathrm{m}}^{n+1-\gamma}}{\gamma\Delta t}=-\frac{1}{\beta}\nabla\cdot(\mathbf{D}_{\mathrm{e}}\nabla\Phi_{\mathrm{e}}^{n+1})-I_{\mathrm{stim}}. \tag{S18}$$

step 3: Integrate the nonlinear reaction part by the BE method

$$C_{\mathrm{m}}\frac{V_{\mathrm{m}}^{n+1}-W_{\mathrm{m}}^{n+1}}{\Delta t/2}=-I_{\mathrm{ion}}(V_{\mathrm{m}}^{n+1},\mathbf{q}^{n+1}), \tag{S19}$$

$$\frac{\mathbf{q}^{n+1}-\mathbf{q}^{n+1/2}}{\Delta t/2}=\mathcal{M}(V_{\mathrm{m}}^{n+1},\mathbf{q}^{n+1}). \tag{S20}$$

2 NUMERICAL RESULTS

The detailed refinement of a coarser grid to get a finer grid and the numbering principle of the triangle grid are shown in Figure S1.

2.1 Numerical Results on a square and cube

Using the variant of the FitzHugh-Nagumo (Vfhn) model, we first give the membrane potential obtained from the CBDF2 scheme at discrete times with a grid of $128\times128\times128$ cells. Figures S2 and S3 show a few iso-contours of membrane potential at different transverse planes at discrete times where the fibers are parallel to the x-axis, *i.e.*, $\mathbf{e}_{\mathrm{l}}=(1,0,0)^T$, while Figure S4 shows several intuitive iso-surfaces of membrane potential at discrete times. We then consider a more complicated situation that the conductivity tensor is space dependent. To be specific, the fibers are counterclockwise around the line $\frac{x+0.5}{-1}=\frac{y+0.5}{-1}=\frac{z+0.5}{1}$, *i.e.*, the muscle sheets are aligned normal to the direction $(-1,-1,1)^T$. Figure S5 shows several iso-surfaces of membrane potential at discrete times.

Figures S6 (S7) shows the iso-contours of membrane voltage collected at different discrete times and a trajectory of membrane voltage collected at a spatial point obtained from the CBDF2 scheme using the more realistic DiFrancesco and Noble (DFN) model and the Courtemanche et al. (CRN) model in the two-dimensional (three-dimensional) space. Due to the limitation of space, only the results with the CBDF2 scheme are presented.

2.2 Numerical Results on a circle and sphere

The computational domain is not limited to the regular areas, *e.g.*, after refining the coarsest grid three times, we can partition the circular region into a quasi-uniform triangle grid by a proper principle as shown in Figure S8. With the increase of the refinement times, the partition grid will converge to the circular region. We first consider a circular region as the computational domain with a center at position (0,0) and a radius of 1. Figure S9 shows the propagating of action potentials obtained from the CBDF2 scheme while Figure S10 shows the trajectories of membrane potentials at different locations both using the Vfhn membrane model.

We then consider a solid sphere (Everett, 2010; Liu and Joe, 1996) as the computational domain with a center at position (0,0,0) and a radius of 0.5. As shown in Figures S11-S13, we present a

few iso-contours and iso-surfaces of membrane potentials at different transverse planes at discrete times obtained from the CBDF2 scheme with the number of smoothing iterations $\nu_{\text{Multigrid}} = 12$.

REFERENCES

Everett, M. E. (2010). A three-dimensional spherical mesh generator. Geophysical Journal of the Royal Astronomical Society 130, 193–200

Liu, A. and Joe, B. (1996). Quality local refinement of tetrahedral meshes based on 8-subtetrahedron subdivision. Mathematics of Computation of the American Mathematical Society 65, 1183–1200

Qu, Z. and Garfinkel, A. (1999). An advanced algorithm for solving partial differential equation in cardiac conduction. IEEE Transactions on Biomedical Engineering 46, 1166–1168

Sundnes, J., Lines, G. T., and Tveito, A. (2005). An operator splitting method for solving the bidomain equations coupled to a volume conductor model for the torso. Mathematical biosciences 194, 233–248

Trangenstein, J. A. and Kim, C. (2004). Operator splitting and adaptive mesh refinement for the luo–rudy i model. Journal of Computational Physics 196, 645–679

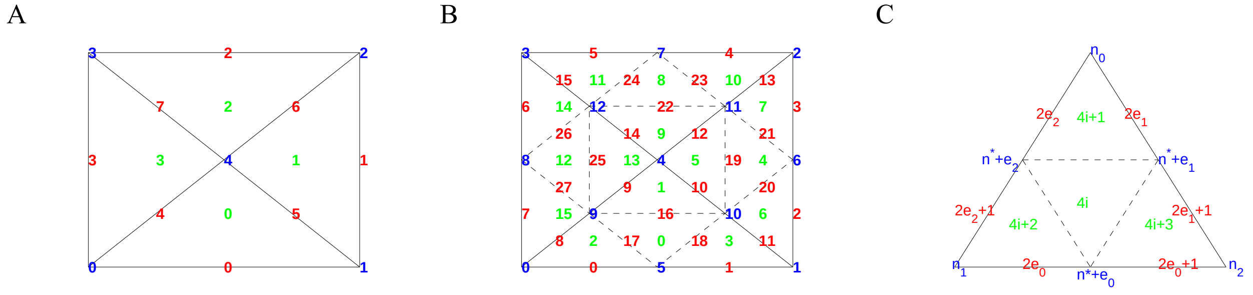

Figure S1. Refinement of the triangular grid. (A) Indices of the nodes (blue), edges (red), and cells (green) of a coarser grid. (B) Indices of the nodes, edges, and cells after cutting each coarser element into four parts. (C) Indices of nodes, edges, and cells of the finer elements from the i-th coarser element where the total number of the coarser grid are denoted by n^*, the indices of edges and nodes of the i-th element in the coarser grid is denoted by e_j and n_j, respectively, for $j = 0, 1, 2$.

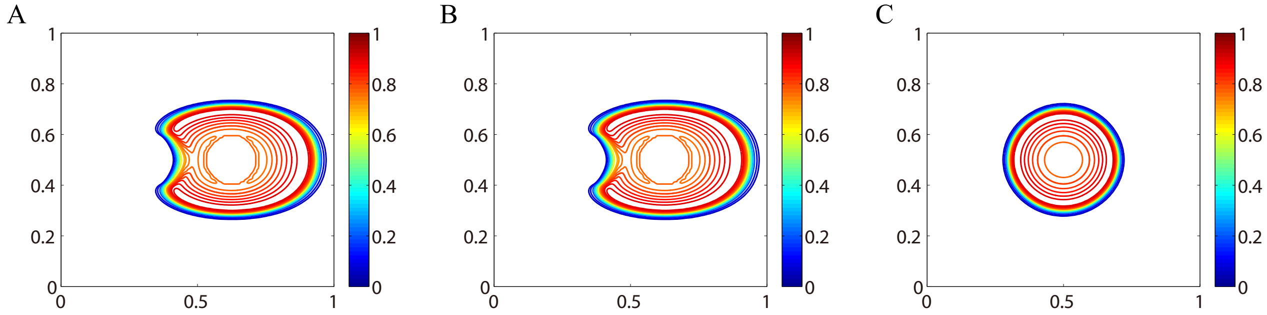

Figure S2. Iso-contours of the membrane potentials at different transverse planes at time $T = 1.0$. (A) the (x, y)-plane with $z = 0.5$; (B) the (x, z)-plane with $y = 0.5$; (C) the (y, z)-plane with $x = 0.5$. The membrane model is the Vfhn model.

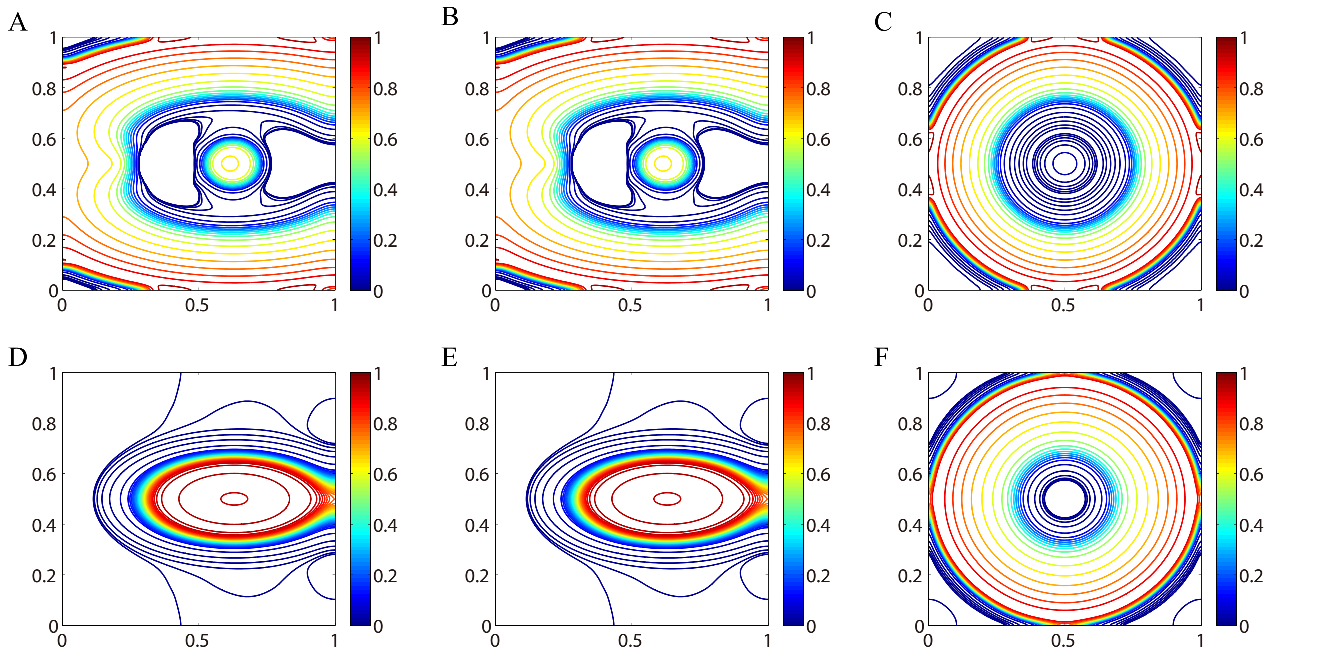

Figure S3. Iso-contours of the membrane potentials at different transverse planes at time $T = 3.0$. (A) The (x, y)-plane with $z = 0.5$; (B) The (x, z)-plane with $y = 0.5$; (C) The (y, z)-plane with $x = 0.5$; (D) The (x, y)-plane with $z = 1.0$; (E) The (x, z)-plane with $y = 1.0$; (F) The (y, z)-plane with $x = 1.0$. The membrane model is the Vfhn model.

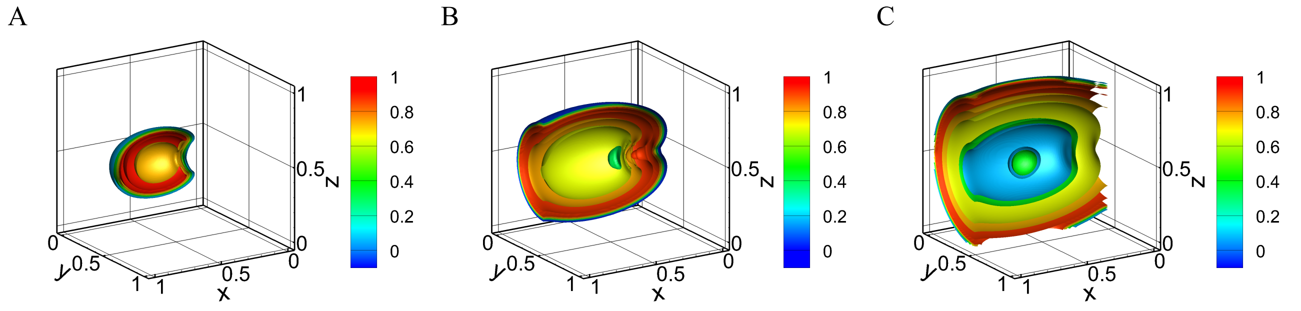

Figure S4. Iso-surfaces of the membrane potentials at different discrete times $T = 1.0$, $T = 2.0$, $T = 3.0$ from left to right. The observing domain is limited with $y \geq 0.5$ and the fibers are parallel to the x axis. The membrane model is the Vfhn model.

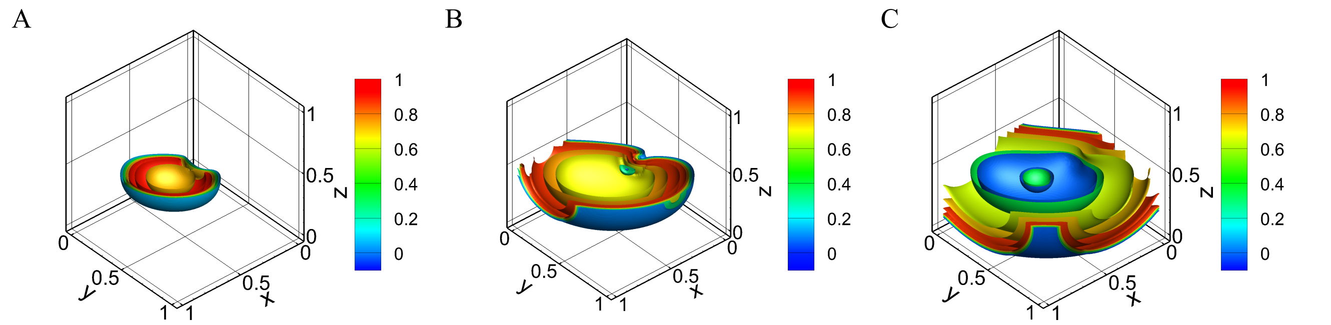

Figure S5. Iso-surfaces of the membrane potentials at different discrete times $T = 1.0$, $T = 2.0$, $T = 3.0$ from left to right. The observing domain is limited with $z \leq 0.5$ and the fibers are set to be space dependent. The grid has $128 \times 128 \times 128$ cells of equal size. The membrane model is the Vfhn model.

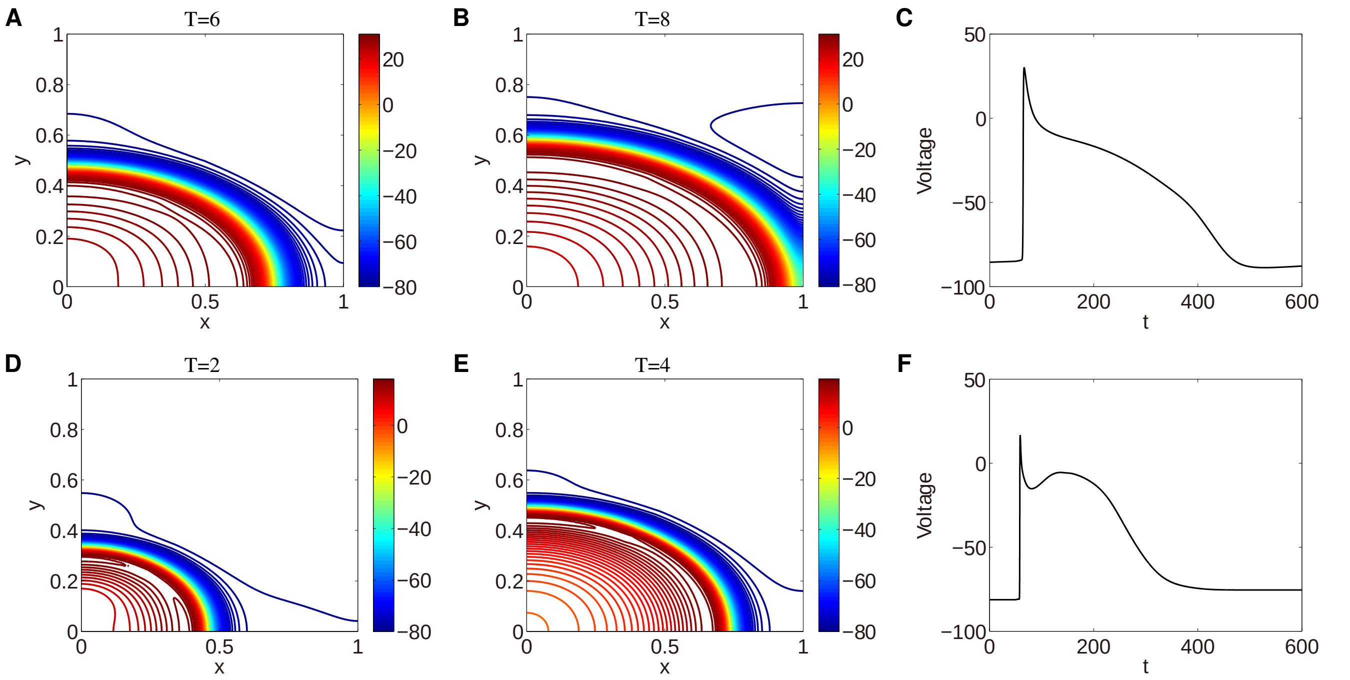

Figure S6. Iso-contours and trajectory of the membrane voltage obtained from the CBDF2 scheme with the DFN membrane model (top panel) and the CRN membrane model (bottom panel) in the two-dimensional space. The spatial step size is $h = 1/32$ and the timestep size is $\Delta t = 1/32$.

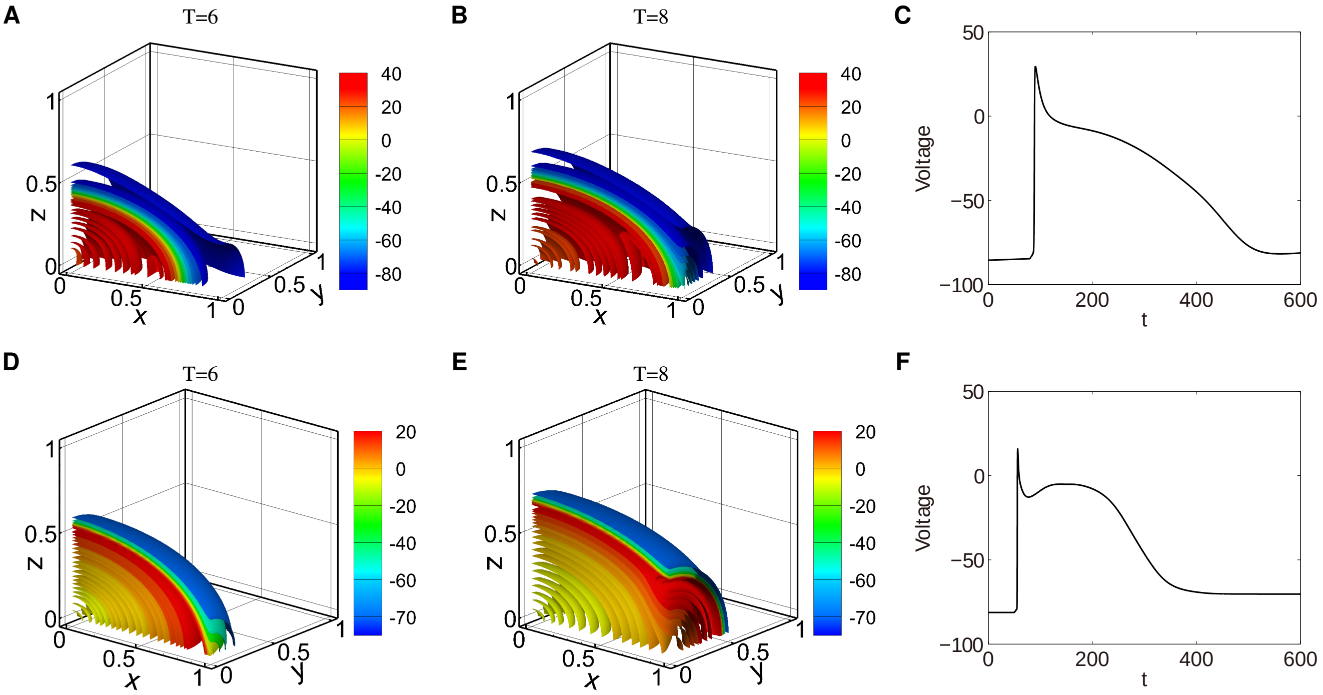

Figure S7. Iso-contours and trajectory of the membrane voltage obtained from the CBDF2 scheme with the DFN membrane model (top panel) and the CRN membrane model (bottom panel) in three-dimensional space. The spatial step size is $h = 1/32$ and the timestep size is $\Delta t = 1/32$.

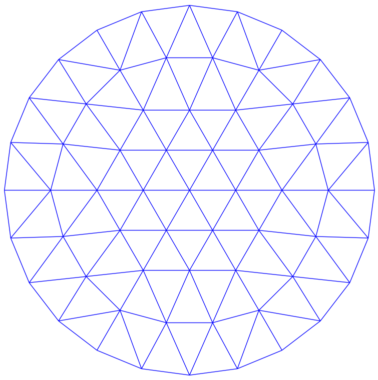

Figure S8. The partition grid of a circular region after refining three times.

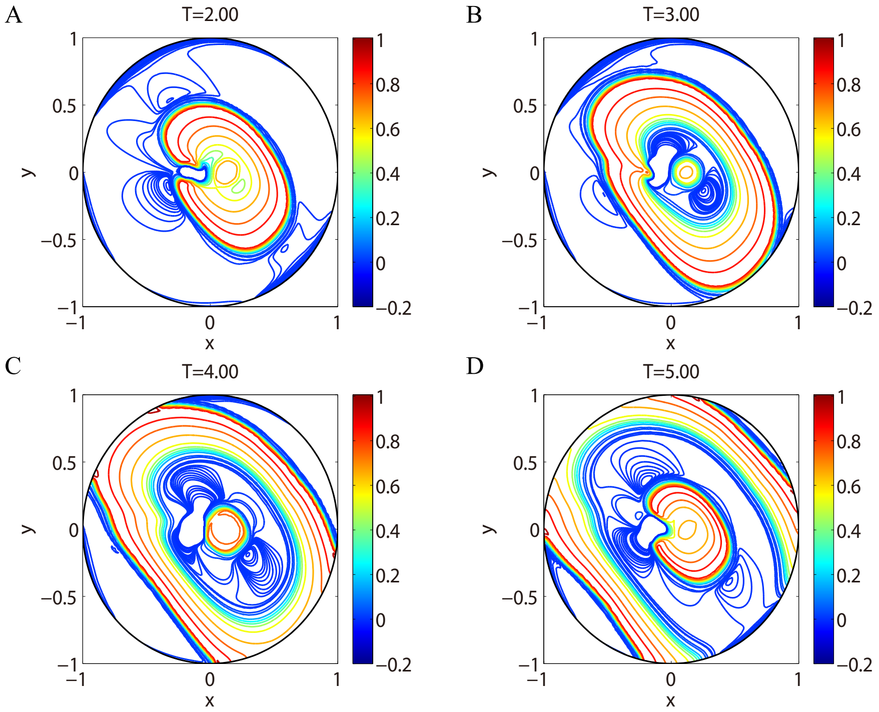

Figure S9. Propagating of action potentials computed by the CBDF2 scheme with timestep size $\Delta t = 1/64$ and spacial step size $h = 1/64$ at discrete times $T = 2.0$, 3.0, 4.0, and 5.0. The membrane dynamics is modeled by the Vfhn model.

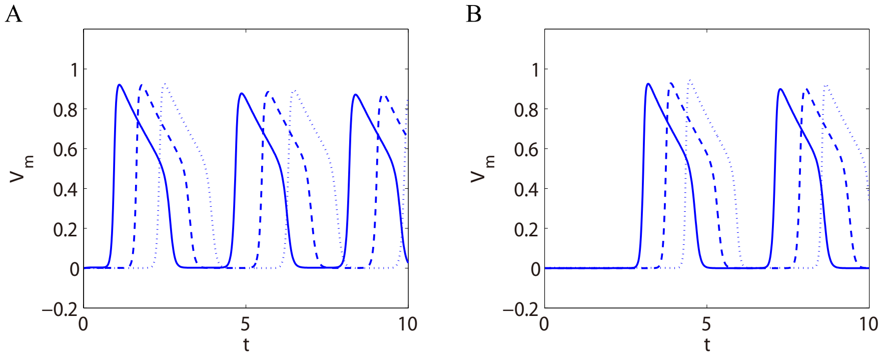

Figure S10. Trajectories of the membrane potentials obtained from the CBDF2 scheme with timestep size $\Delta t = 1/64$ and spacial step size $h = 1/64$ at different positions. (A) The solid, dashed, and dotted lines indicate the membrane potentials collected at position (0.375,0), (0.5,0), and (0.625,0), respectively. (B) The solid, dashed, and dotted lines indicate the membrane potentials collected at position (0.75,0), (0.875,0), and (1,0), respectively. The membrane model is the Vfhn model.

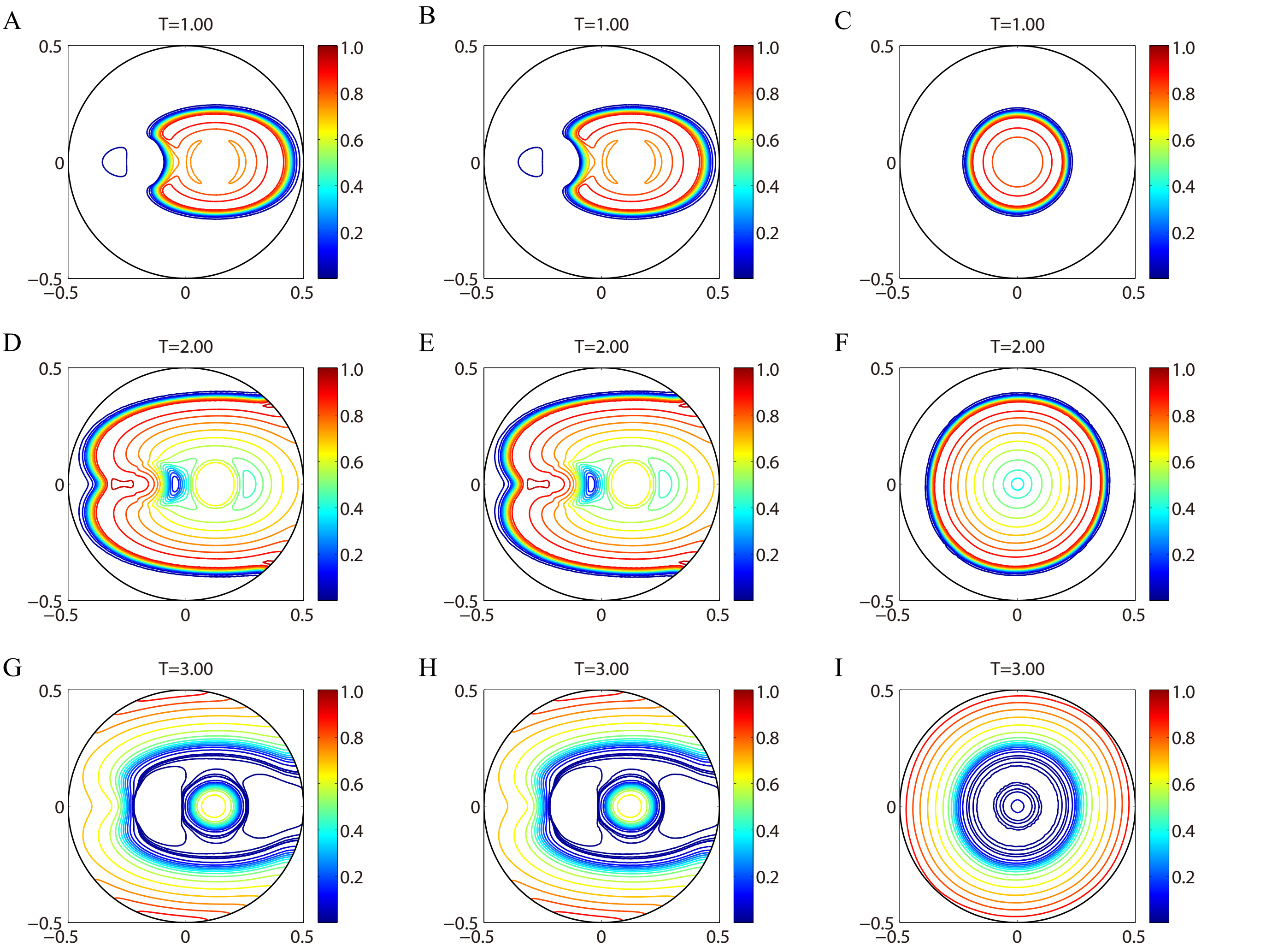

Figure S11. Iso-contours of the membrane potentials obtained from the CBDF2 scheme at different transverse planes at time $T = 1.0$, $T = 2.0$ and $T = 3.0$. The first column: the (x, y)-plane with $z = 0.0$; the second column: the (x, z)-plane with $y = 0.0$; the third column: the (y, z)-plane with $x = 0.0$. The membrane model is the Vfhn model.

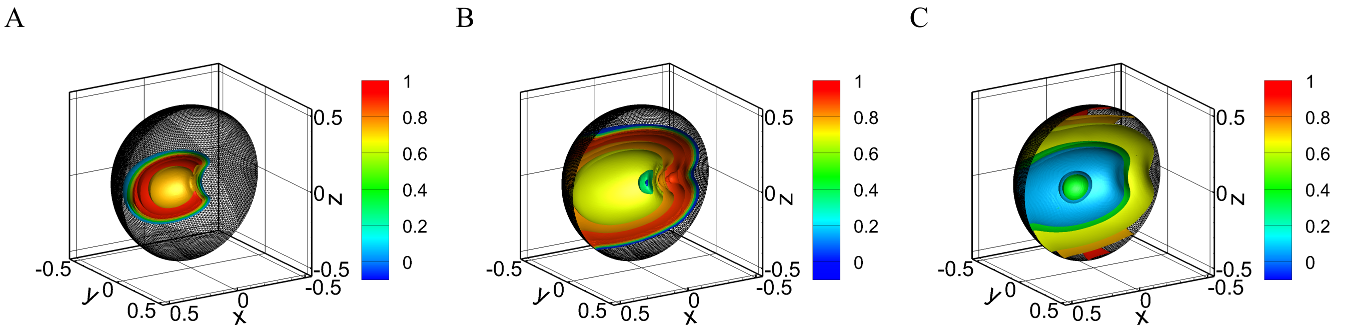

Figure S12. Iso-surfaces of the membrane potentials obtained from the CBDF2 scheme at different discrete times $T = 1.0$, $T = 2.0$, $T = 3.0$ in the domain where $y \leq 0$. The wave is symmetric in the region $y < 0$ and the fibers are parallel to the x axis. The membrane model is the Vfhn model.

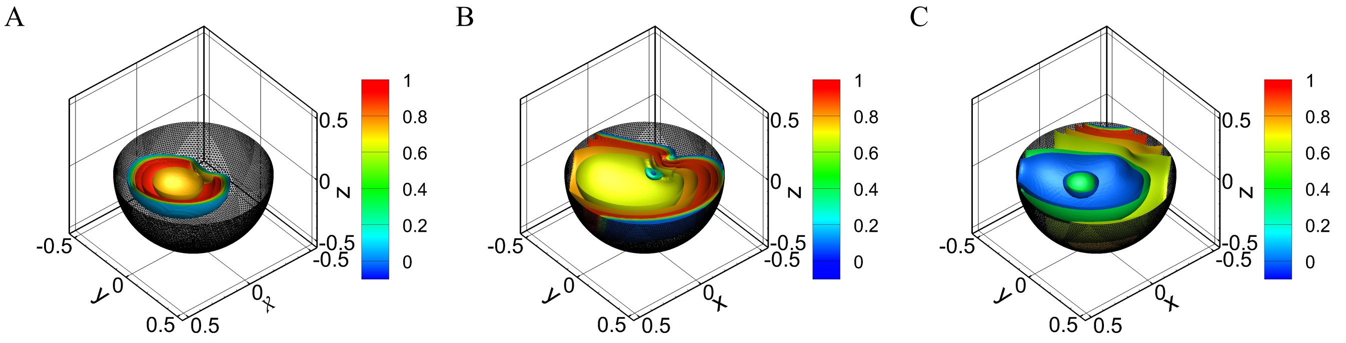

Figure S13. Iso-surfaces of the membrane potentials obtained from the CBDF2 scheme at different discrete times $T = 1.0$, $T = 2.0$, $T = 3.0$ in the domain where $z \leq 0$. The fibers are set to be space dependent. The membrane model is the Vfhn model.
